# A Robust Self-Alignment Method for Ship's Strapdown INS Under Mooring Conditions

**DOI:** 10.3390/s130708103

**Published:** 2013-06-25

**Authors:** Feng Sun, Haiyu Lan, Chunyang Yu, Naser El-Sheimy, Guangtao Zhou, Tong Cao, Hang Liu

**Affiliations:** 1 Marine Navigation Research Institute, College of Automation, Harbin Engineering University, Harbin 150001, China; E-Mails: sunfeng407@126.com (F.S.); chunyangy@yahoo.cn (C.Y.); zhougt@hrbeu.edu.cn (G.Z.); caotongheu@163.com (T.C.); 2 Department of Geomatics Engineering, 2500 University Drive NW, University of Calgary, Calgary, AB T2N 1N4, Canada; E-Mail: elsheimy@ucalgary.ca; 3 National Space Science Center, Chinese Academy of Sciences (CAS), Beijing 100190, China; E-Mail: liuhang_heu@126.com

**Keywords:** inertial navigation systems (INS), alignment, mooring condition, inertial measurement unit (IMU), inertial frame, hidden Markov model, Kalman filter

## Abstract

Strapdown inertial navigation systems (INS) need an alignment process to determine the initial attitude matrix between the body frame and the navigation frame. The conventional alignment process is to compute the initial attitude matrix using the gravity and Earth rotational rate measurements. However, under mooring conditions, the inertial measurement unit (IMU) employed in a ship's strapdown INS often suffers from both the intrinsic sensor noise components and the external disturbance components caused by the motions of the sea waves and wind waves, so a rapid and precise alignment of a ship's strapdown INS without any auxiliary information is hard to achieve. A robust solution is given in this paper to solve this problem. The inertial frame based alignment method is utilized to adapt the mooring condition, most of the periodical low-frequency external disturbance components could be removed by the mathematical integration and averaging characteristic of this method. A novel prefilter named hidden Markov model based Kalman filter (HMM-KF) is proposed to remove the relatively high-frequency error components. Different from the digital filters, the HMM-KF barely cause time-delay problem. The turntable, mooring and sea experiments favorably validate the rapidness and accuracy of the proposed self-alignment method and the good de-noising performance of HMM-KF.

## 1. Introduction

Alignment is the essential procedure for strapdown inertial navigation systems (INS). The purpose of alignment is to establish the initial attitude transformation matrix between the body frame and navigation frame [1]. Before the start of navigation, accurate alignment is crucial for strapdown INS if precise navigation results, namely position, velocity and attitude (PVA), are to be achieved, especially for the ship's strapdown INS which may be required to provide PVA information of a ship with relatively high precision over periods of days, weeks, or even longer [2,3].

Normally, alignment techniques can be categorized into two types based on the conditions of strapdown INS: stationary alignment and in-motion alignment [4–6]. The essence of stationary alignment is to compute the initial attitude matrix using two non-collinear vectors, namely the gravity and the earth rotational rate measurements from accelerometers and gyroscopes respectively [7,8]. Nowadays, the stationary alignment has been studied and developed very well [9–12]. In-motion alignment means alignment on a moving or shaking platform, in this case, different from stationary alignment, the above mentioned gravity and earth rotational rate measurements cannot be accurately measured due to the external motion dynamics of the vehicles. Sometimes, there is not enough time for strapdown INS to get alignment process done on a static platform at the starting point [13–15]. And also strapdown INS applied in ships or weapon systems of vessels have to move in response to the motion of the sea and wind waves both under mooring and voyaging conditions [16–18]. Moreover, after starting the navigation calculation procedure, re-alignment process is necessary for strapdown INS on the moving platform, as the errors of navigation solution could gradually grow up due to the poor initial alignment inaccuracies and the growing sensor errors [19–21]. Accordingly, it is essential to explore the reliable in-motion alignment schemes for strapdown INS.

**Figure 1.**
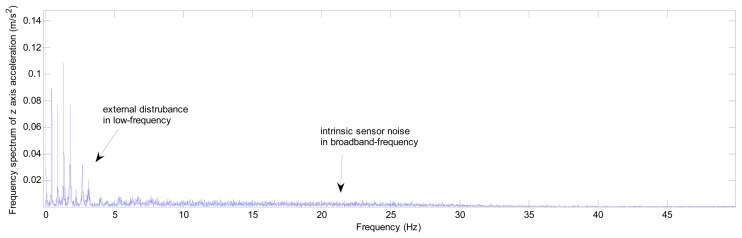
The z axis acceleration frequency spectrum under mooring condition.

The alignment of ship's strapdown INS contains mooring alignment in docks and alignment at sea based on the conditions of the ship [17]. As for alignment at sea, the auxiliary external information should be used, such as position information provided by the global positioning system (GPS) [22–28], velocity reference given by the Doppler velocity log (DVL) [29–31], *etc.* This article will not discuss the methods of alignment at sea. Mooring alignment belongs to in-motion alignment. As mentioned above that under mooring condition, the ship has to withstand external random movements which are mainly caused by the sea wind and sea waves, and mostly the frequencies of these external movements are higher than 1/15 Hz [32]. So the primary problem of ship's strapdown INS alignment under mooring condition is that the gravity and the earth rotational rate measurements will be disturbed by lineal and angular movements of the ship especially for the accelerations measurements, thus resulting in the alignment accuracy falling and time increasing [16–18]. Also, as state-of-the-art inertial measurement unit (IMU) employed in a strapdown INS often suffers from intrinsic sensor noise, especially for the low-end tactical grade and low-cost IMU, thus the alignment angles more or less may not be computed accurately and rapidly enough [21,22]. Figure 1 shows the frequency spectrum of the z axis raw acceleration of a ship's strapdown INS under mooring condition, The INS contains a self-made medium level IMU, and its sampling frequency is 100 Hz (the relative coordinate frames definition and experimental details will be addressed further in this article). It can be depicted that the raw measurements of z axis accelerometer are mainly mixed with both the intrinsic sensor noise components (in broadband-frequency) and the relatively low-frequency error components which are mostly caused by the external disturbance. Besides, other inertial sensors' outputs of this IMU have the same frequency spectrum characteristic as the outputs of the z axis accelerometer [17].

In this work, we concentrate on the investigation of mooring alignment method for ship's strapdown INS. As is evident from the above discussions, how to extract the useful gravity and angular rate of earth rotation measurements from the IMU outputs is the primary objective. Two useless error components which are blended in the useful measurements need to be removed as much as possible before or during the alignment process:

1. Intrinsic sensor noise.
2. External random disturbance.

For solving this problem, studies on alignment of ship's strapdown INS under mooring conditions have employed various approaches. The conventional Kalman filter methods or other optimal estimation methods are utilized in [13,24–26] to estimate the misalignment angles. However, as the estimation process is affected by the external disturbed movements of the ship, the alignment duration will be beyond 20 minutes [20]. In some studies, the pre-filters are designed to remove or attenuate the influence of external disturbed movements and sensor noise. Wavelet de-noising technique is used in [21,22] to remove the high-frequency sensors noise components. Lian *et al.* [33,34] firstly give the idea of utilizing the finite impulse response (FIR) digital filters to attenuate disturbing accelerations, and they claim this method is suitable for large initial misalignment angles cases. Similarly, Zhao [35,36], Zhang [37] and Li, *et al.* [38] utilize the cascaded FIR filters. However, as proved by Zhang in [37], these FIR filters should be designed with very large orders to meet the de-nosing requirement, thus resulting in the increasing of both the computation burden and alignment time. In [39], we adopt the infinite impulse response (IIR) digital low-pass filter to process the gyroscope and accelerometer outputs. The proposed IIR filter can reduce well the influence caused by the disturbed movements of the ship with very small filter orders. Nevertheless, one major drawback of all these digital filters is that they will more or less suffer from time-delay problems in real-time implementations. Lü *et al.* [40,41] propose a novel prefitler which combines a Kalman filter and a IIR digital filter to filter the outer disturbances and the sensor noise in a real-time way, different from the Wavelet de-noising techniques, which have block processing structures, the computation time of the frefilter is much shorter than the Wavelet methodology. Gaiffe [42] and Napolitano [43] give the idea of inertial frame based alignment (IFBA) scheme which uses the projection of gravity in the inertial frame to calculate the attitude matrix between inertial frame and body frame. This scheme can attenuate the disturbed movements, and quickly obtain the initial attitude matrix. Various authors have presented solutions using this method [44–50]. As analyzed in [49], most of the periodical and low-frequency random disturbance can be removed by the mathematical integration and average characteristic of this method, however, the relatively high-frequency and non-periodical components would stay all through the alignment process.

The Hidden Markov model (HMM) is a powerful statistical tool for the modelling of sequence data [51]. It has been originally applied to stochastic signal processing, and nowadays has been widely used in speech recognition [52,53], human movement analysis [54], *etc.* Enlightened by Lü *et al.* [40,41] and the theory of HMM [51], we consider the useful IMU outputs for alignment as a HMM, viz., the valid measurements of IMU used for INS alignment (the local gravity and earth rotational rate) are hidden in the IMU's raw outputs which include intrinsic sensor noise and external disturbance. Based on this, we propose a two-dimensional hidden Markov model based Kalman filter (HMM-KF) to pre-process the IMU output signals, most of the error components referred above could be filtered out by the proposed HMM-KF, then the useful data for INS alignment can be obtained. Different from the pre-filters in [17,21,22,33–39], the main advantage of the proposed HMM-KF is that the processing of sequential data will barely cause time-delay problem. After pre-processing the IMU outputs, useful input measurements are used in the following alignment process which can be divided into two steps *i.e.*, coarse alignment and fine alignment [5]. Due to the benefits that the IFBA method can counteract and average the low-frequency periodical disturbed accelerations, in our approach, we adopt the IFBA method, both in the coarse and fine alignment processes.

The remainder of this paper is organized as follows: Section 2 addresses the coordinate frames used in this paper. Section 3 describes the modified IFBA method for ship's INS under mooring condition. Section 4 introduces the principle of the proposed two-dimensional HMM-KF, its usefulness for de-noising the error components of the IMU outputs is evaluated by experiments. Section 5 details the connection between HMM-KF and low-pass digital filter, which explains why the HMM-KF can remove the high-frequency noise components, and time-delay performance comparison will be shown using the HMM-KF as compared with the corresponding low-pass digital filter. Experimental results that validate the proposed approach are presented and discussed in Section 6. Finally, conclusions are given in Section 7.

## 2. Frames Definitions

**Figure 2.**
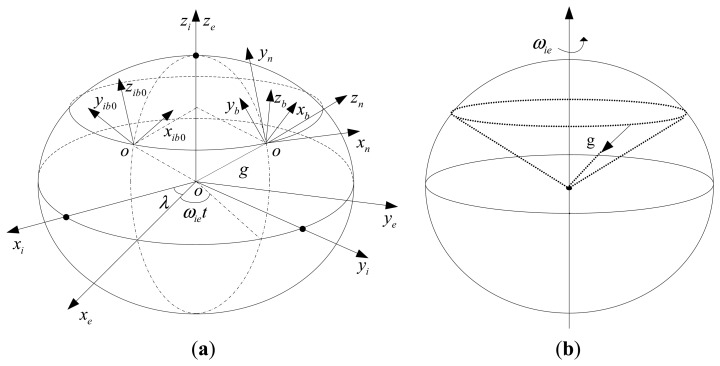
(**a**) The different coordinate frames; (**b**) Conical movements of the gravity vector in inertial frame.

The different coordinate frames used in this paper are defined as follows and illustrated in Figure 2(a). Each frame is an orthogonal, right-handed coordinate frame.

(1) The *e* frame: Earth-fixed frame, with its z axis paralleling the earth's rotation axis, x and y axes are fixed and parallel equatorial plane.
(2) The *i* frame: Inertial frame, stabilized in inertial space, at the beginning of inertial alignment, it parallels earth frame.
(3) The *n* frame: Navigation frame, used for navigation and attitude representation, in this work, we choose the local level coordinate frame as the *n* frame, its x, y and z axis point to local east, north and upward respectively.
(4) The *b* frame: Body frame, IMU sensor coordinate frame with its origin at the centroid of IMU, three axes parallel inertial sensors' input axes.
(5) The *i*_*b*0_ frame: Body inertial frame, at the beginning of inertial alignment procedure, it is formed by fixing the *b* frame in the inertial space.

## 3. Modified Inertial Frame based Alignment Method

### 3.1. Problem Formulation

The traditional analytical alignment of strapdown INS is to calculate the initial value of attitude matrix $C_{b}^{n}$ between the *b* frame and the *n* frame by using the following equation [3]:

(1) $$C_{b}^{n}={\left[\begin{array}{c} {\left[g^{n}\right]}^{T}\\ {\left[\omega_{ie}^{n}\right]}^{T}\\ {\left[g^{n}\times \omega_{ie}^{n}\right]}^{T} \end{array}\right]}^{-1}\left[\begin{array}{c} {\left[f^{b}\right]}^{T}\\ {\left[\omega^{b}\right]}^{T}\\ {\left[f^{b}\times \omega^{b}\right]}^{T} \end{array}\right]$$

where *gn*=[0 0 *- g*] *^T^* and $\omega_{ie}^{n}={\left[0\;\omega_{ie}\;\cos L\;\omega_{ie}\sin L\right]}^{T}$ are the projections of the local gravity vector *g* and the turn rate of the earth *ω_ie_* in the *n* frame respectively, *L* is the local latitude, *f^b^* and *ω^b^* both can be easily extracted from the measurements of IMU (see e.g., [3] page 25-57 for details) are the projections of *g* and *ω_ie_* on the *b* frame respectively. This analytical method is conditional upon the precondition that the strapdown INS is on a static platform during the alignment process. As for ship's strapdown INS under mooring condition, due to the external movements of the ship, the IMU outputs are mixed with external random disturbance, so the alignment method mentioned above is inapplicable.

### 3.2. Inertial Frame based Alignment

As we know that the pure gravity vector can form a cone in the inertial frame due to the rotation of the Earth, see Figure 2(b). So the projections of gravity vector in the inertial frame change slowly with a period of 24 hours, namely the Earth's rotation period. When the ship's strapdown INS is under mooring conditions, most of the disturbed angular rate and acceleration components vary periodically and in relatively low frequencies [44]. By projecting the measurements of accelerometers and gyroscopes into the inertial frame, the periodic disturbed components can be averaged and counteracted, the pure gravity vector and earth rotational rate vector remain [45,46]. Given the particularity of ship's strapdown INS under mooring condition and by making full useful of the information mentioned above, here we give the derivation of the analytical inertial frame based alignment method for ship's strapdown INS. This method contains coarse and fine alignment process, and both are derived in the inertial frame.

#### 3.2.1. Coarse Alignment

In order to fulfill the alignment process, the body inertial frame (the *i*_*b*0_ frame) is introduced firstly, which is formed by fixing the *b* frame in the inertial space at the beginning of the alignment (*t*_0_), namely $C_{b}^{i_{b0}}(t_{0})=I$. Then after the start of alignment, the *i*_*b*0_ frame is fixed in the inertial space. So the alignment matrix between the *b* frame and the *n* frame can be expressed by:

(2) $$C_{b}^{n}=C_{i}^{n}C_{i_{b0}}^{i}C_{b}^{i_{b0}}$$

where $C_{b}^{i_{b0}}$ represents the attitude transformation matrix of the strapdown INS body frame (*b* frame) relative to the *i*_*b*0_ frame, and can be instantaneously updated by using the following expression [3]:

(3) $${\dot{C}}_{b}^{i_{b0}}=C_{b}^{i_{b0}}[\omega_{ib}^{b}\times ]$$

where $\omega_{ib}^{b}\times$ is the skew symmetric matrix of the vector $\omega_{ib}^{b}={\left[\omega_{\text{ibx}}^{b},\omega_{\text{iby}}^{b},\omega_{\text{ibz}}^{b}\right]}^{T}$ measured by the gyroscopes represents the turn rate of the *b* frame with respect to the *i* frame [3]. Notice that the external disturbed angular velocity *δω^s^* and the random error of gyroscopes are included in $\omega_{ib}^{b}$.

Also, the matrix $C_{i}^{n}$ can be updated by a function of local latitude *L* and the time *t* as follows:

(4) $$C_{i}^{n}=\left[\begin{array}{ccc} -\sin[\omega_{ie}(t-t_{0})] & \cos[\omega_{ie}(t-t_{0})] & 0\\ -\sin L\cos[\omega_{ie}(t-t_{0})] & -\sin L\sin[\omega_{ie}(t-t_{0})] & \cos L\\ \cos L\cos[\omega_{ie}(t-t_{0})] & \cos L\sin[\omega_{ie}(t-t_{0})] & \sin L \end{array}\right]$$

As the mooring alignment is generally implemented in the specific docks, the local latitude *L* in Equation (4) is a known quantity. $C_{i_{b0}}^{i}$ is a constant matrix representing the transformation matrix between the *i* frame and the *i*_*b*0_ frame, the detailed derivation of $C_{i_{b0}}^{i}$ can be seen in Appendix A.

Finally, substituting for $C_{b}^{i_{b0}}$, $C_{i}^{n}$ and $C_{i_{b0}}^{i}$ respectively from Equations (3), (4) and (A8) in Equation (2) gives the coarse alignment result $C_{b}^{n}$. Worth noting that in Equation (A4), the accelerometer sensor noise component is ignored, and in Equation (3), the gyroscope outputs $\omega_{ib}^{b}$ used to update $C_{b}^{i_{b0}}$ contain sensor noise. Moreover, after the integration of Equation (A5), the non-periodic disturbance components remain, so the further fine alignment stage is indispensable.

#### 3.2.2. Fine Alignment

We use the standard Kalman filter to estimate the misalignment angles in the fine alignment procedure, the state and measurement equations of the Kalman filter are both established in the inertial frame. The velocity-error differential equation and the misalignment angles equation in the inertial frame could be written as:

(5) $$\{\begin{array}{l} \delta {\dot{V}}^{i}=-g^{i}\times \varphi^{i}+C_{b}^{i}\nabla_{\text{bias}}^{b}+C_{b}^{i}\nabla_{\text{noise}}^{b}\\ {\dot{\varphi }}^{i}=-C_{b}^{i}{ɛ}_{\text{bias}}^{b}-C_{b}^{i}{ɛ}_{\text{noise}}^{b} \end{array}$$

where $\delta V^{i}={\left[\delta V_{x}^{i}\;\delta V_{y}^{i}\;\delta V_{z}^{i}\right]}^{T}$ is the velocity-error vector in inertial frame. *g^i^* is given in Equation (5), $\varphi^{i}={\left[\varphi_{x}^{i}\;\varphi_{y}^{i}\;\varphi_{z}^{i}\right]}^{T}$ denotes the misalignment angle vector. $\nabla_{\text{noise}}^{b}$ and ${ɛ}_{\text{noise}}^{b}$ are Gaussian white noises related to the velocity errors and the misalignment angles respectively. $\nabla_{\text{bias}}^{b}$ is the random acceleration bias vector, ${ɛ}_{\text{bias}}^{b}$ is the random gyroscope drift vector, both are assumed in gauss distribution, namely ${\dot{\nabla }}_{\text{bias}}^{b}=0$, ${\dot{ɛ}}_{\text{bias}}^{b}=0$. The detailed derivation procedure can be seen in [45].

Equation (5) can be regarded as the standard linear system driven by the Gaussian white noises. Then the state equation of the fine alignment Kalman filter can be represented as:

(6) $$\dot{X}=\left[\begin{array}{llll} 0_{3\times 3} & -[g^{i}\times ] & 0_{3\times 3} & C_{b}^{i}\\ 0_{3\times 3} & 0_{3\times 3} & -C_{b}^{i} & 0_{3\times 3}\\ 0_{3\times 3} & 0_{3\times 3} & 0_{3\times 3} & 0_{3\times 3}\\ 0_{3\times 3} & 0_{3\times 3} & 0_{3\times 3} & 0_{3\times 3} \end{array}\right]X+\left[\begin{array}{llll} C_{b}^{i} & 0_{3\times 3} & 0_{3\times 3} & 0_{3\times 3}\\ 0_{3\times 3} & -C_{b}^{i} & 0_{3\times 3} & 0_{3\times 3}\\ 0_{3\times 3} & 0_{3\times 3} & 0_{3\times 3} & 0_{3\times 3}\\ 0_{3\times 3} & 0_{3\times 3} & 0_{3\times 3} & 0_{3\times 3} \end{array}\right]\left[\begin{array}{c} {\left[\nabla_{\text{noise}}^{b}\right]}_{3\times 1}\\ {\left[{ɛ}_{\text{noise}}^{b}\right]}_{3\times 1}\\ 0_{3\times 1}\\ 0_{3\times 1} \end{array}\right]$$

where $X={\left[{\left[\delta V^{i}\right]}^{T}{\left[\varphi^{i}\right]}^{T}{\left[{ɛ}_{\text{bias}}^{b}\right]}^{T}{\left[\nabla_{\text{bias}}^{b}\right]}^{T}\right]}^{T}$ is the state vector. The measurement equation is formulated as:

(7) $$Z=\left[\begin{array}{c} {\hat{V}}_{x}^{i}-V_{x}^{i}\\ {\hat{V}}_{y}^{i}-V_{y}^{i}\\ {\hat{V}}_{z}^{i}-V_{z}^{i} \end{array}\right]=[\begin{array}{cc} I_{3\times 3} & 0_{3\times 9} \end{array}]X+N_{3\times 1}$$

where *^N^*_3×1_ is the measurement noise vector, $V^{i}={\left[V_{x}^{i}\;V_{y}^{i}\;V_{z}^{i}\right]}^{T}$ given in Equation (A2) is the integration of the gravity in the inertial frame, ${\hat{V}}^{i}={\left[{\hat{V}}_{x}^{i}\;{\hat{V}}_{y}^{i}\;{\hat{V}}_{z}^{i}\right]}^{T}$ is the velocity of the strapdown INS calculated in the inertial frame, and is given by:

(8) $${\hat{V}}^{i}=\int C_{b}^{i_{b0}}f^{b}dt$$

Equations (6) and (7) constitute the state and measurement model respectively. Through using the discrete Kalman filter, the converging values of the misalignment angles could be estimated, then the coarse attitude matrix derived in Equation (2) will be corrected. Although all the remained components depicted in the last section are in small quantities, however, the alignment speed slows down due to these error components, also the precision of alignment results are still more or less affected by these components [17]. If precise and rapid alignment results are to be achieved, these error components should be pre-filtered as much as possible. In next section, a novel technique is proposed to solve this problem.

## 4. Hidden Markov Model Based Kalman Filter

**Figure 3.**
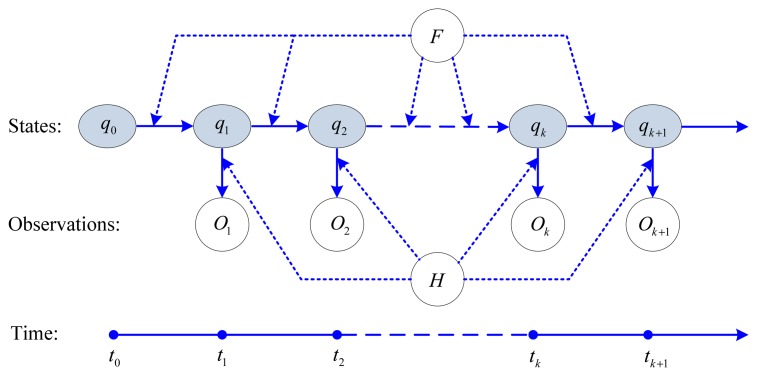
Topology structure of the hidden Markov model.

In an ordinary Markov model, the states are directly visible to the observations. However, in a hidden Markov model (HMM), the states are not directly visible, but the outputs dependent on the states are visible. Each state has a probability distribution over the possible output sequence. Therefore the output sequence generated by the HMM gives some information about the sequence of the states. Figure 3 shows the topology structure of the HMM.

In Figure 3, {*O_k_*} is the observation sequence at time step *t_k_*, {*qk*} in the shadow circle denotes the hidden state sequence, and *q_0_* is the initial value of the state. *H* is the transition matrix between the hidden state {*q_k_*} and observation {*Ok*}, *F* is the transition matrix between the states *q*, both *H* and *F* are governed by probability distributions.

### 4.1. HMM of IMU Outputs

We define *X_k_* (*k* = 1,2,…) as the IMU ideal output sequence (a Markov chain) which is valid for INS alignment. When the ship is under mooring condition, *X_k_* is disturbed by the additive sensor noise and random movements of the ship, so the resultant output sequence is *Z_k_*. From the preceding discussion we know that *X_k_* is hidden in *Z_k_* (particularly when *X_k_* = *Z_k_*, the state is no longer hidden) then the HMM can be formulated as follows:

(9) $$X_{k+1}=FX_{k}+\mu_{k+1}$$

(10) $$Z_{k+1}=HX_{k}+v_{k+1}$$

where Equations (9) and (10) are discrete in the time, in the state space and the measurement space respectively, *X* ∈ *R^N^* , *Z* ∈ *R^M^* , the matrices *F* and *H* consist of transition probabilities and have elements *F_ij_*, *H_ij_*, satisfying:

(11) $$\sum_{j=1}^{N}F_{ij}=1,\sum_{j=1}^{M}H_{ij}=1,F_{ij},H_{ij}\geq 0$$

In this study the IMU outputs are modeled as a two dimensional HMM. And based on the characteristics of IMU outputs, Equations (9) and (10) can respectively be expanded into [55]:

(12) $$\left[\begin{array}{c} X_{k+1}\\ X_{k} \end{array}\right]=\left[\begin{array}{cc} 1 & 0\\ 0 & 1 \end{array}\right]\;\left[\begin{array}{c} X_{k}\\ X_{k-1} \end{array}\right]+\left[\begin{array}{c} \mu_{k+1}\\ \mu_{k} \end{array}\right]$$

(13) $$\left[\begin{array}{c} Z_{k+1}\\ Z_{k} \end{array}\right]=\left[\begin{array}{cc} 1 & 0\\ 0 & 1 \end{array}\right]\;\left[\begin{array}{c} X_{k}\\ X_{k-1} \end{array}\right]+\left[\begin{array}{c} v_{k+1}\\ v_{k} \end{array}\right]$$

We term *μ_k_* the driving noises and *ν_k_* the measurement noise, they are assumed to be zero mean Gaussian white noise, satisfying:

$$\begin{array}{c} E\{\mu_{i}\}=0,cov\{\mu_{i},\mu_{j}\}=E\{\mu_{i},\mu_{j}^{T}\}=Q_{i}\delta_{ij}\\ E\{v_{i}\}=0,cov\{v_{i},v_{j}\}=E\{v_{i},v_{j}^{T}\}=R_{i}\delta_{ij}\\ cov\{\mu_{i},v_{j}\}=E\{\mu_{i},v_{j}^{T}\}=0 \end{array}$$

### 4.2. Kalman Filter Based on HMM

After deriving the discrete HMM, the next step is to estimate the state sequence *X_k_* from the measurement sequence *Z_k_* using some optimal algorithms. An optimal solution is through combining the standard Kalman filter with HMM to remove the noise and other perturbation components [51]. The standard Kalman filter equations are described in [56], in each filtering step, the Kalman gain *K* is calculated and used to correct the propagated state when a measurement is available. In this specific application, the dynamics matrix *F* and measurement matrix *H* of HMM can be regarded as constant matrices, so the Kalman gain *K* will quickly tend to be a constant, represented by *K_0_*. Then the Kalman filter equations based on the two dimensional HMM are simplified as follows:

- Computing the state prediction:
  (14) $$\left[\begin{array}{l} {\hat{X}}_{k+1/k}\\ {\hat{X}}_{k/k} \end{array}\right]=F_{k,k-1}\left[\begin{array}{l} {\hat{X}}_{k}\\ {\hat{X}}_{k-1} \end{array}\right]$$
- Updating the state estimate:
  (15) $$\left[\begin{array}{l} {\hat{X}}_{k+1}\\ {\hat{X}}_{k} \end{array}\right]=\left[\begin{array}{l} {\hat{X}}_{k+1/k}\\ {\hat{X}}_{k/k} \end{array}\right]+K_{0}\left(\left[\begin{array}{l} {\tilde{X}}_{k+1}\\ {\tilde{X}}_{k} \end{array}\right]-H_{k}\left[\begin{array}{l} {\hat{X}}_{k+1/k}\\ {\hat{X}}_{k/k} \end{array}\right]\right)$$
- Smoothing the two state estimate for removing the filtering ripples:
  (16) $$X_{k+1}^{*}=({\hat{X}}_{k+1}+{\hat{X}}_{k})/2$$
  where *X˜*_*k*+1_ is the IMU output at time step *k* + 1, $X_{k+1}^{*}$ is the smoothed estimate at time step *k* + 1.

### 4.3. HMM-KF Implementation Experiments

The proposed HMM-KF algorithm was applied to the real data collected from a self-made medium-accuracy IMU. The IMU consists of three interferometric fiber optic gyroscopes (IFOG) and three accelerometers mounted in three mutually orthogonal directions. The specifications of the self-made IMU are shown in Table 1. During the experiments, the IMU was on a mooring ship, so the error components of the IMU outputs under this condition mainly contain the external disturbance and sensor noise. The HMM-KF parameters of accelerometer outputs were initially set as follows:

$$Q_{a}=\text{diag}{\left\{0.003*10^{-3}g,\;0.003*10^{-3}g\right\}}^{2},\;R_{a}=\text{diag}{\left\{100*10^{-4}g,\;100*10^{-4}g\right\}}^{2}$$

where *Q_a_* and *R_a_* are the process noise covariance and measurement error covariance of the filter, *g* is the local gravity. Similarly, the HMM-KF initial parameters of gyroscope *Q_g_* and *R_g_* are:

$$Q_{g}=\text{diag}{\left\{0.5*10^{-5}({}^{\circ}/h),\;0.5*10^{-5}({}^{\circ}/h)\right\}}^{2},\;R_{g}=\text{diag}{\left\{50*10^{-5}({}^{\circ}/h),\;50*10^{-5}({}^{\circ}/h)\right\}}^{2}$$

**Figure 4.**
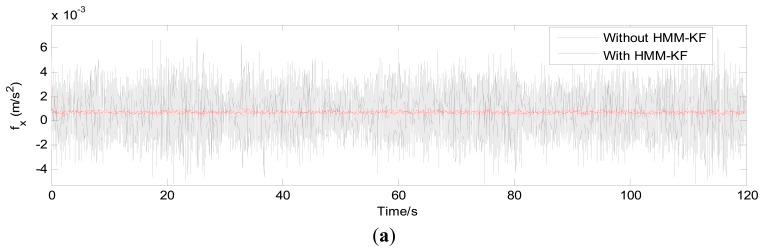

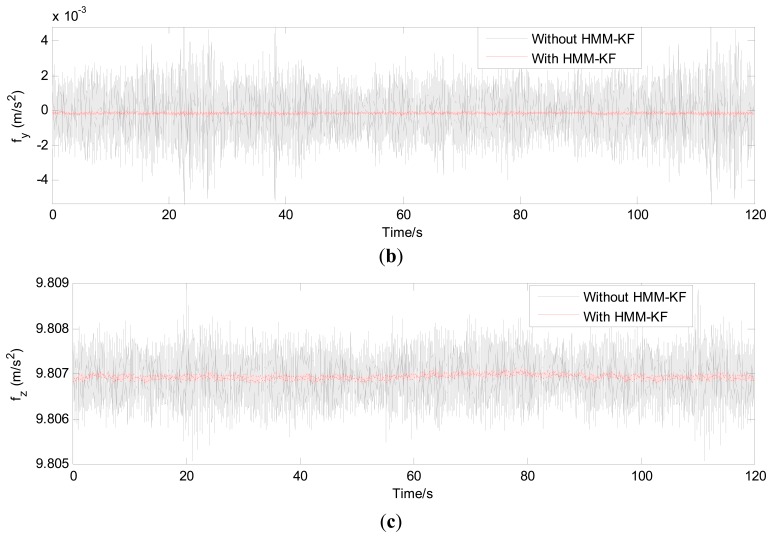
Time-domain analysis of the accelerometer outputs with and without HMM-KF (**a**) x axis accelerometer; (**b**) y axis accelerometer; (**c**) z axis accelerometer.

**Figure 5.**
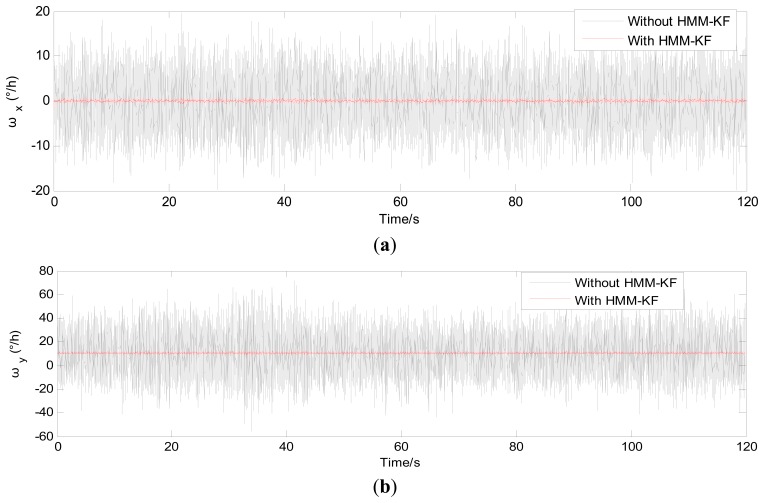

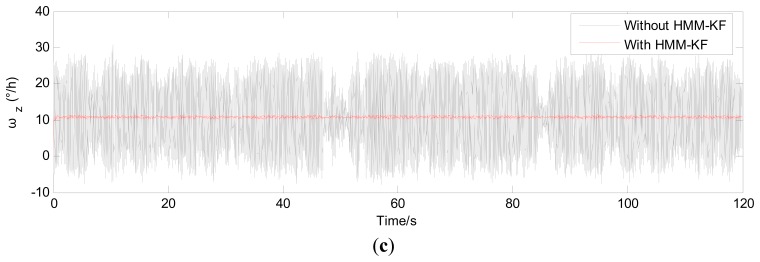
Time-domain analysis of the gyroscope outputs with and without HMM-KF. (**a**) x axis gyroscope; (**b**) y axis gyroscope; (**c**) z axis gyroscope.

The choices of the parameters *Q_a_*, *R_a_*, *Q_g_*, *R_g_* will be discussed in the following section. Figures 4 and 5 respectively show the accelerometer and gyroscope outputs in time-domain with and without using the proposed HMM-KF. Table 2 and Table 3 list the standard deviation (STD) of the error components of each inertial sensor for both the original and the filtered outputs.

**Figure 6.**
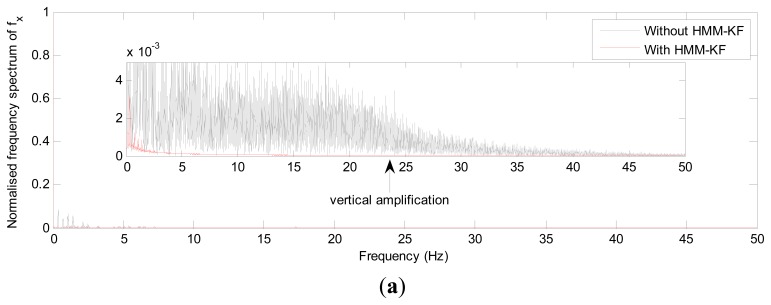

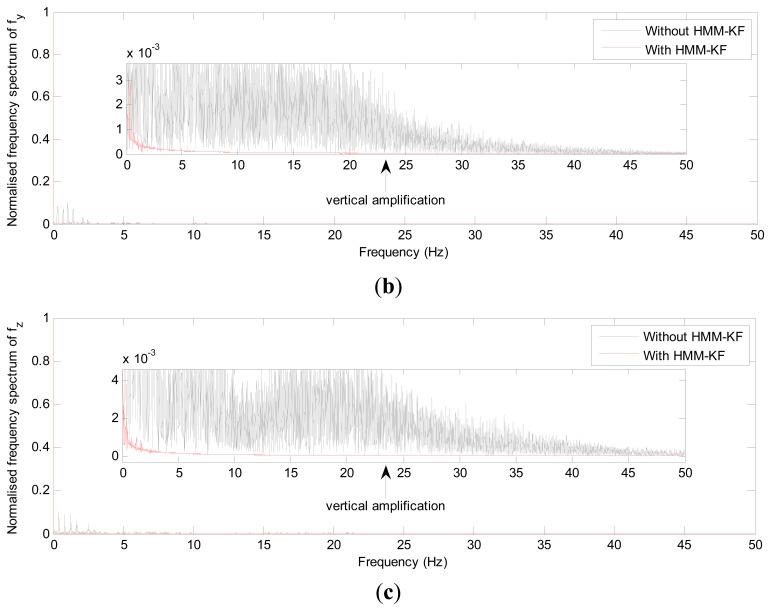
Frequency-domain analysis of the accelerometer outputs with and without HMM-KF (**a**) x axis accelerometer; (**b**) y axis accelerometer; (**c**) z axis accelerometer.

**Figure 7.**
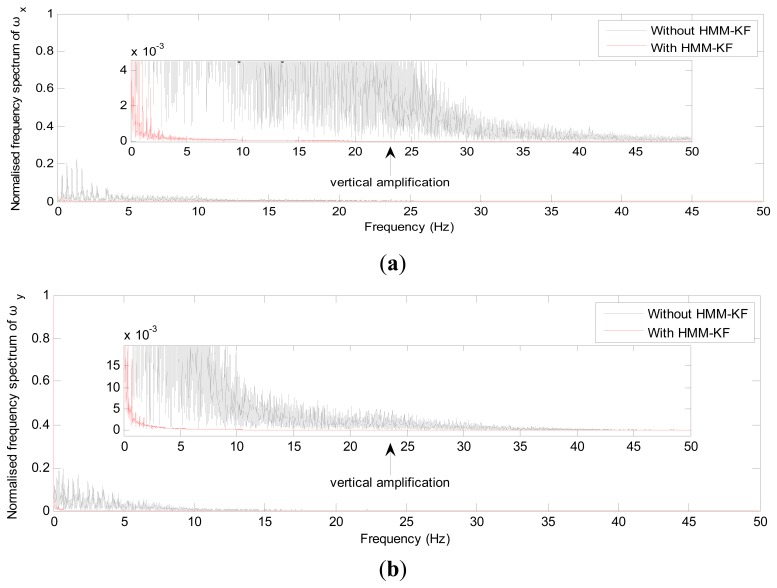

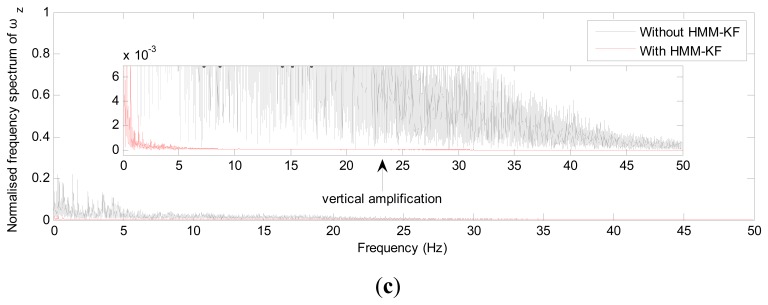
Frequency-domain analysis of the gyroscope outputs with and without HMM-KF (**a**) x axis gyroscope; (**b**) y axis gyroscope; (**c**) z axis gyroscope.

It is obvious from Figures 4 and 5 that most of the noise components are removed. Tables 2 and 3 provide the comparison of the standard deviation of the accelerometer and gyroscope outputs with and without using the proposed HMM-KF. The two tables indicate that significant reductions in noise components are achieved through using the HMM-KF. Figures 6 and 7 respectively analyze the accelerometer and gyroscope outputs in frequency-domain with and without the HMM-KF. It can be depicted that the HMM-KF can provide obvious attenuation of noise components with specific frequency bands for both the accelerometer and gyroscope outputs.

## 5. Analyses of HMM-KF

In this section, some discussions on the principle of the proposed HMM-KF are introduced, which elaborate the de-noising characteristic of the HMM-KF. By mathematical derivation, we found the filtering property of the HMM-KF, as it can be changed to the form of digital filter's difference equation. Then experiments were conducted to compare the HMM-KF with the corresponding digital filter. Results clearly show that the proposed HMM-KF has better real-time characteristic as compared with the corresponding digital filter, while the digital filter gets obvious signal time delay(s) under the same filtering effect.

### 5.1. Connections between Digital Filter and the HMM-KF

Substituting Equation (14) in Equation (15), we have:

(17) $${\hat{X}}_{k}=(I-K_{0}H_{k})F_{k,k-1}{\hat{X}}_{k-1}+K_{0}Z_{k}$$

Substituting Equations (12) and (13) in Equation (17), expanding it yields:

(18) $$\left[\begin{array}{c} X_{k+1}\\ X_{k} \end{array}\right]=\left(\left[\begin{array}{cc} 1 & 0\\ 0 & 1 \end{array}\right]-K_{0}\cdot \left[\begin{array}{cc} 1 & 0\\ 0 & 1 \end{array}\right]\right)\cdot \left[\begin{array}{cc} 1 & 0\\ 0 & 1 \end{array}\right]\cdot \left[\begin{array}{c} X_{k}\\ X_{k-1} \end{array}\right]+K_{0}\cdot \left[\begin{array}{c} Z_{k+1}\\ Z_{k} \end{array}\right]$$

Simplifying it, one gets:

(19) $$\{\begin{array}{l} X_{k+1}=(1-K_{0})\cdot X_{k}+K_{0}\cdot Z_{k+1}\\ X_{k}=(1-K_{0})\cdot X_{k-1}+K_{0}\cdot Z_{k} \end{array}$$

Assume that *x* = *Z*, *y* = *X*, then the Equation (19) can be described as the form:

(20) $$y(k+1)=(1-K_{0})\cdot y(k)+K_{0}\cdot x(k+1)$$

We infer from Equation (20) that the value of *y* at time *k* + 1 only depends on the current input *x*(*k* + 1) and the prior output *y*(*k*). This propagation model is the standard recursive digital filter's difference equation [57]. So Equation (20) formulates that *the HMM-KF has the digital filter's characteristics, which explains why HMM-KF can remove the noise and error components of the IMU outputs. Moreover, the connection between HMM-KF gain *K*_0_ and the cutoff frequency *f_c_* of the corresponding digital filter is as follows:

(21) $$f_{c}=\frac{1}{2\pi {\left(1/f_{s}\right)}^{2}}\cdot \arccos\frac{2-2K_{0}-{\left(K_{0}\right)}^{2}}{2(1-K_{0})}$$

where *f_s_* is the sampling frequency, and the detailed derivation procedures are given in Appendix B. Through using Equation (21), the HMM-KF and the corresponding digital filter can be mutually transformed.

Commonly, before using digital filters for signals processing, the signals' frequency spectrum or power spectrum should be analysed in advance, based on this, the filter's cutoff frequency *f_c_* can be determined. In contrast to the use of digital filters, the initialization of HMM-KF is relatively easier. As the choices of the initial measurement error covariance matrix *R* and the process noise covariance matrix *Q* are less deterministic in the actual implementation of Kalman filter [56], this is also suitable for the proposed HMM-KF. But once *Q* and *R* are determined, HMM-KF gain *K*_0_ will stabilize quickly and then remain constant. To our knowledge, the different initial conditions of *Q* and *R* for the HMM-KF do not influence the filters' performances clearly, this will be evaluated and discussed next in the experiments part of this section.

### 5.2. Comparisons of the HMM-KF and the Corresponding Digital Filters

Once *Q* and *R* are determined off-line, we can get the deterministic HMM-KF gain *K*_0_, then the corresponding digital filter's cutoff frequency *f_c_* can be determined by Equation (21). Two different digital filters have been widely used: IIR (Infinite Impulse Response) filters and FIR (Finite Impulse Response) filters. In general, IIR filters could be approximated by a prescribed frequency response with relatively fewer multiplications and lower computation burdens than FIR filters, because that the FIR filters need much higher orders than the corresponding IIR filters to meet the same filtering requirements. However, the FIR filters are capable of working with a strict linear-phase, *i.e.*, the time delay between the inputs and outputs of FIR filters can be exactly known [57]. For this reason, IIR filters are more reliable in the applications that do not need real-time requirements and are in low computation abilities, while the FIR filters are always employed in systems which need to know the accurate filtering time delay. Here in our study, both the corresponding IIR and FIR low-pass digital filters were designed and analyzed to compare the real-time abilities and the filtering performances with the proposed HMM-KF.

The comparison experiments were conducted through using the same IMU data as depicted in Section 4.3. In this work, we use the *MATLAB*/*Filter Design & Analysis Tool* to design the two digital filters. The sampling frequency of the IMU is 100 Hz, then *F_s_* equals 100 Hz. For the IIR filters, we choose the Butterworth low-pass digital filters and specify the filter order *N_IIR_* as 5 and 10 respectively. For the FIR filters, the transition-band is set as [*f_c_*–0.05 Hz, *f_c_*+0.05 Hz], the pass-band attenuation *A_p_* is 1 *dB*, while the stop-band attenuation *A_s_* equals 40 *dB*, 60 *dB* respectively. Worth noting that in order to reduce the FIR filters' orders for the convenience of giving the time-delay comparisons, we intentionally set the transition-band of the FIR filters relatively in width.

**Figure 8.**
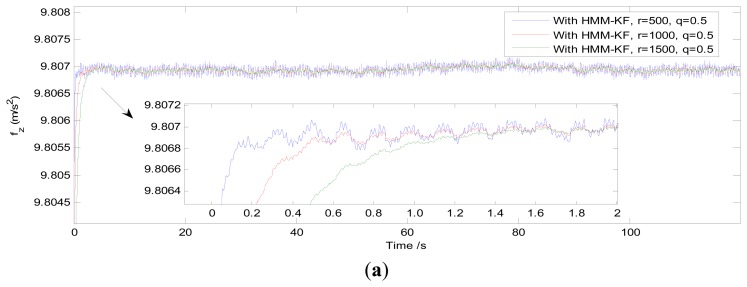

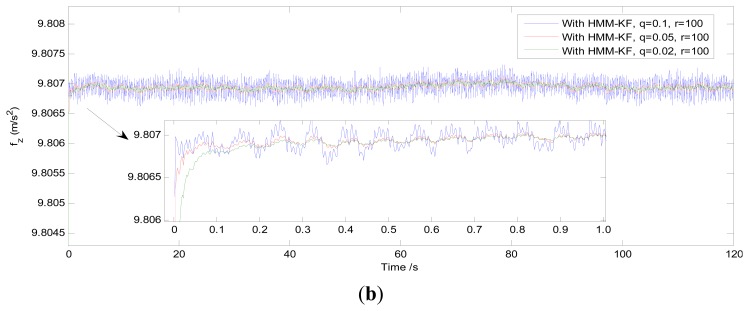
Comparison of the HMM-KF filtering results using the different parameters in Table 4. (**a**) *q* remains 0.5, *r* = 500, 1,000, 1,500; (**b**) *r* remains 100, *q* = 0.1, 0.05, 0.02.

Assume that the different initial parameters *Q_a_*, *R_a_*, *Q_g_*, *R_g_* of HMM-KF are expressed by:

$$\begin{array}{c} Q_{a}=\text{diag}{\left\{q\cdot 10^{-3}g,\;q\cdot 10^{-3}g\right\}}^{2},\;R_{a}=\text{diag}{\left\{r\cdot 10^{-4}g,\;r\cdot 10^{-4}g\right\}}^{2}\\ Q_{g}=\text{diag}{\left\{m\cdot 10^{-5}({}^{\circ}/h),\;m\cdot 10^{-5}({}^{\circ}/h)\right\}}^{2},\;R_{g}=\text{diag}{\left\{n\cdot 10^{-5}({}^{\circ}/h),\;n\cdot 10^{-5}({}^{\circ}/h)\right\}}^{2} \end{array}$$

where *q*, *r*, *m* and *n* are variables, Table 4 gives the different values of *K*_0_, *f_c_* and *N_FIR_* (minimum order of FIR in different stop-band attenuation *A_p_*) for *q* remains 0.5, *r* = 500, 1,000, 1,500; and for *r* remains 100, *q* = 0.1, 0.05, 0.02. Similarly, Table 5 shows the corresponding values of *K*_0_, *f_c_* and *N_FIR_*, when *m* and *n* respectively remain constants or change as variables. Figure 8 provides the HMM-KF filtering results of the z axis acceleration using the different parameters *q* and *r* in Table 4.

**Figure 9.**
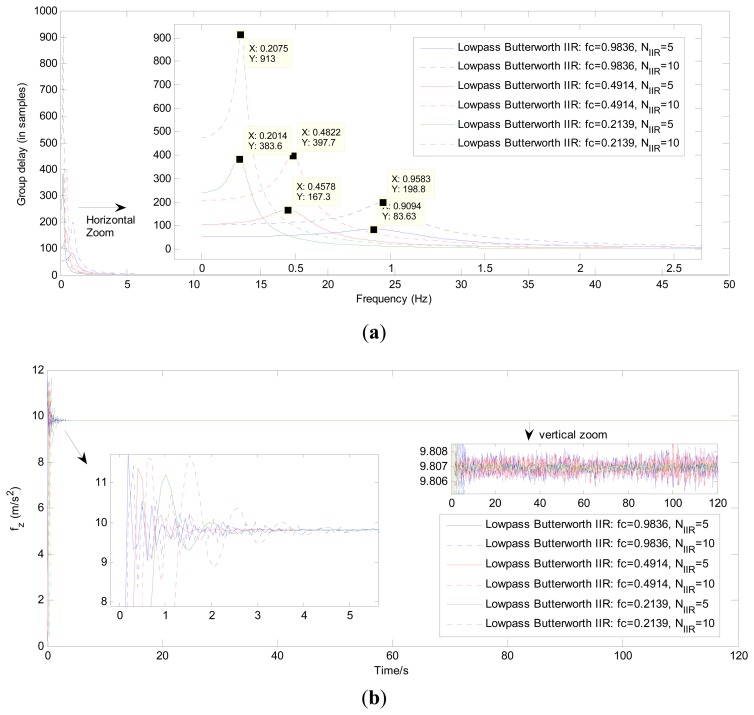
(**a**) Group time delay of IIR filters with different *f_c_* and orders in broad-band frequencies; (**b**) IIR filtering results of the z axis acceleration with different *f_c_* and orders.

Figure 9 compares the group time delay and the filtering results of the IIR filters using the different parameter *f_c_* and filter order *N_IIR_*.

**Figure 10.**
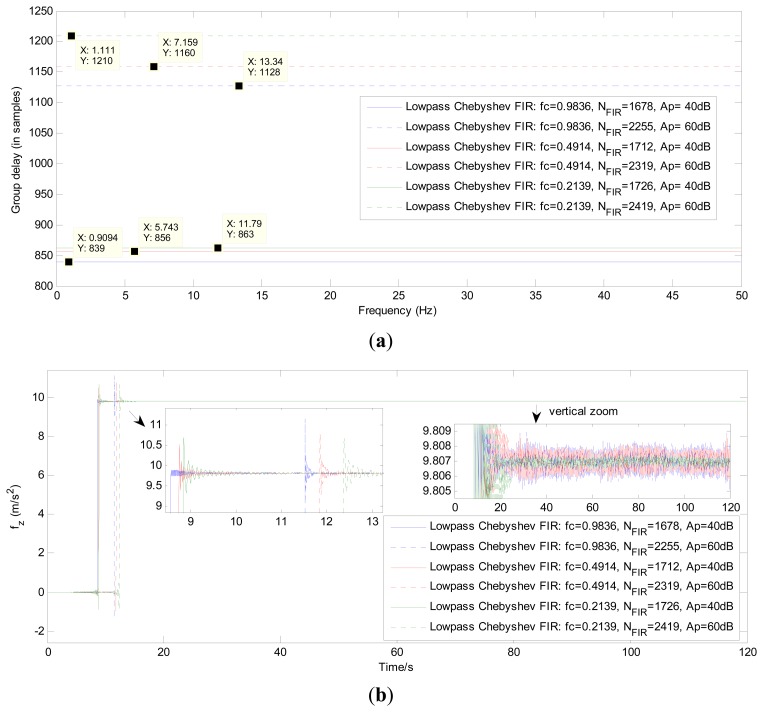
(**a**) Group time delay of FIR filters with different *f_c_* and *A_s_* in broad-band frequencies; (**b**) FIR filtering results of the z axis acceleration with different *f_c_* and *A_s_*.

Figure 10 gives the exact group time delay and the filtering results of the FIR filters using the parameters *f_c_* and *N_FIR_* in Table 4. Tables 6 and 7 present the results of the time delay (in samples) after using the three different approaches to process the z axis accelerometer outputs and z axis gyroscope outputs respectively.

Without reference to the time-delay issue, it can be seen in Figure 8 and the vertical-zoomed pictures of both Figures 9(b) and 10(b) that the de-noising performance of the proposed HMM-KF has almost the same level as compared with the other two digital filter approaches. And the de-noising performance of the three methods can be achieved and gradually increased through decreasing both the values of HMM-KF gain *K*_0_ and the digital filter's cutoff frequency *f_c_*. However, increasing the de-noising performance of the three filters will more or less cause the time-delay problem. And there are significant differences in the time-delay performance of the three different approaches. The smaller the values of *K*_0_ and *f_c_* are, the larger the time delay of the three filters will get, obviously for the IIR and FIR digital filters, slightly for the proposed HMM-KF, that can be seen in Figures 7, 8, 9 and 10, and summarized in Tables 6 and 7. Some conclusions on the time-delay issue of the three approaches are given as follows:

1. As above mentioned that the input-output of the IIR filters do not satisfy linear-phase, so the time delay of the IIR filters cannot be exactly calculated and given. Also, it can be seen in Figure 9(a) that the blended signal components with frequencies around cutoff frequency *f_c_* suffer from relatively larger time delay than that with frequencies not close to *f_c_*.
2. As for the FIR digital filters, it is shown in Tables 4 and 5 that even with relatively wide transition-bands, the minimum orders of the FIR filters are much higher than the corresponding IIR filters. Moreover, the larger the values of *A_p_* are, the larger the orders of the FIR filters will be, thus resulting in obvious time delay, which can be seen in Figure 10(b). However, as shown in Figure 10(a) that for each specific FIR filter, the time delay could remain a certain constant after processing signals with any frequency bands. And, the time delay of the specific FIR filter can be exactly calculated using the following equation [57]:
  (22) $$T_{\text{delay}}=\frac{N_{\text{FIR}}-1}{2F_{s}}$$
3. As for the HMM-KF, it can be depicted in Figures 8 and 9 (drawing the z axis acceleration de-noising results) that effects of lowering the values of *q* and meanwhile keeping the values of *r* in a constant will barely cause the time-delay problem. Conversely, the HMM-KF could suffer from obvious time-delay problem to a certain extent when *q* remains 0.5, *r* varies in 500, 1,000 and 1,500.

So we can experimentally conclude that under the same de-noising performance, the proposed HMM-KF has better real-time abilities than the digital filters. And how to make the HMM-KF work appropriately depends on the adjusting of the values of parameters *Q* and *R*. As different IMU have different noise levels, the optimization of choosing the values of *Q* and *R* cannot be mathematically solved. But it is no doubt that the HMM-KF parameters *Q* and *R* impact not only the de-noising performance of the IMU outputs, but also the filtering response speed, namely the time-delay level. The de-noising performance increases together with the values of parameters *R*, while the time delay increase slightly for the HMM-KF. Conversely, the de-nosing performance increases together with the decrease of the values of *Q*, while the time delay barely increase for HMM-KF. So in real-time implementations, in order to augment the filtering performance at no cost of increasing the time delay, the parameter *Q* should be kept in relatively small values, and through adjusting the values of *R* to meet the good de-noising performance. In addition, the conclusions addressed above are equivalent to every channel of the inertial sensors, *i.e.*, the three accelerometers and the three gyroscopes, as the relevant experiments have been conducted through using the measurements of all these sensors.

Under static conditions, the time-delay problem does not affect the INS alignment results very much, whereas under mooring or voyaging conditions, as the INS are sometimes in the dynamic modes, errors caused by the time delay could accumulate gradually all through the alignment process [6]. This is will be validated and discussed in Section 6. Firstly, two different alignment mechanisms using the proposed HMM-KF, FIR and IIR filters will be given and discussed in the following section.

### 5.3. Alignment Mechanisms Using Different Filters

In Section 5.2, the time-delay performance of the proposed HMMKF and digital filters were compared and discussed. In order to cope with the time-delay issue in the alignment process when using the different pre-filters, two alignment mechanisms are given in this section.

**Figure 11.**
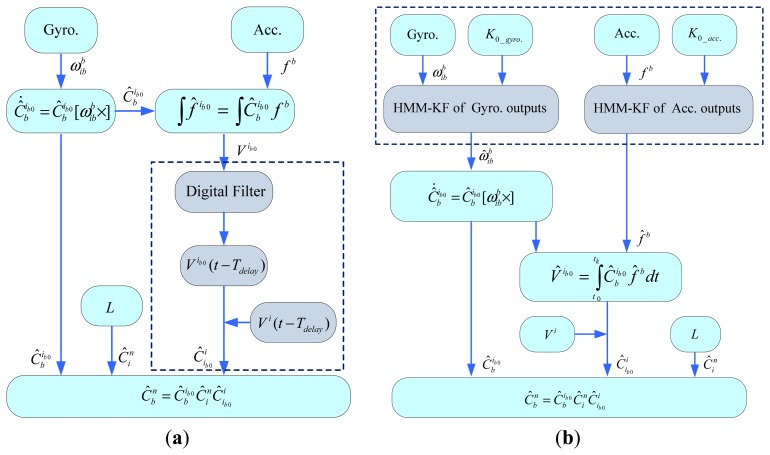
Flowcharts of the digital filter aided and the HMM-KF aided inertial frame based alignment mechanisms. (**a**) Digital filter aided; (**b**) HMM-KF aided.

According to references [42] and [45] that the inertial frame based alignment method can well attenuate the angular-motion disturbances, and also for avoiding the situation that the time delay of the accelerometer's digital filter may not be identical to that of the gyroscope's digital filter, in this way, the digital filters are employed only to remove the linear-motion disturbances and thus extracting the pure gravity for the INS alignment. The flowchart of the digital filter aided inertial frame based alignment mechanism can be seen in detail in Figure 11(a). As specified in ref. [33], the digital filters should be adopted after the integration of specific force *f^b^* , then the output of the digital filter *V*^*ib*0^ at current time step *t* corresponds to the value *V*^*ib*0^ (*t*–*T_delay_*) at a former time step *t*–*T_delay_* with the time delay *T_delay_*. In order to eliminate the effect of the time delay *T_delay_* and still get the correct value of $C_{i_{b0}}^{i}$ derived from Equation (A8), equally, the value of *V^i^* calculated using Equation (A2) at current time step *t* should be represented by *V^i^* (*t*–*T_delay_*) at time step *t*–*T_delay_*.

For each specific FIR filter, as *T_delay_* can be accurately calculated using Equation (22), the alignment results will not be influenced by the FIR filters very much, however the alignment duration and computation burden can be greatly increased, because as interpreted in Section 5.2, *T_delay_* is always in a very large value. For the IIR digital filters, *T_delay_* cannot be exactly given, but relatively is in small value, so sometimes the time delay of IIR filters used in the application of INS alignment is ignored, such as the schemes given by Sun, *et al.* in [17] and Yan, *et al.* in [44].

Different from the digital filter aided inertial frame based alignment mechanism, the HMM-KF aided mechanism does not have to consider the time-delay issue, which can be avoided through adjusting the initial parameters *Q* and *R*, both for the outputs of the gyroscope and accelerometer. So the HMM-KF can favorably and properly be used as the optimal pre-filters to pre-process the gyroscope outputs and accelerometer outputs before and during the alignment procedures, the details of the HMM-KF aided mechanism can be seen in Figure 11(b).

## 6. Alignment Experiments and Performance Evaluation

In order to evaluate the performance of the proposed new robust self-alignment method for the ship's strapdown INS under mooring condition, in this section, several experiments under different circumstances were conducted. The turntable experiments were implemented in the lab, as the test condition was relatively ideal and uncomplex, the external disturbances were constrained in periodical forms, so only the coarse alignment process was conducted. In Section 6.2, both the coarse and fine alignment procedures were conducted to test the robust alignment method in the ship's mooring experiments. At last, the data from a sea experiment was used to validate the IFOG online de-noising performance of the proposed HMM-KF in the navigation calculation stage.

### 6.1. Turntable Coarse Alignment Experiments

**Figure 12.**
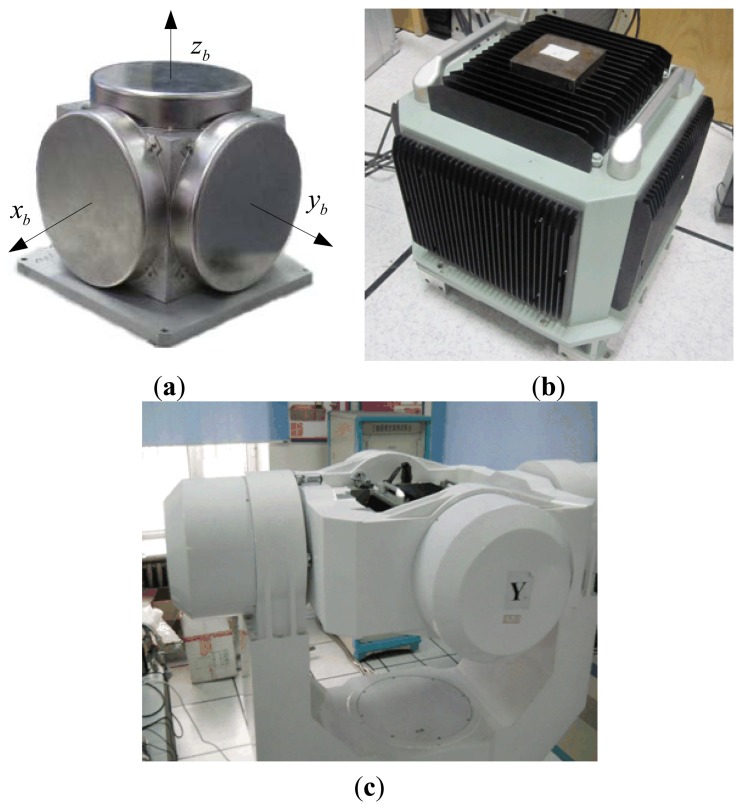
(**a**) Self-made IFOG-IMU and its body frame; (**b**) IFOG-IMU based strapdown INS; (**c**) STG-3 turntable and the experiment scene.

We fixed the IFOG-IMU based strapdown INS which is produced by our laboratory on the SGT-3 three-axis turntable to implement the coarse alignment experiments. The IMU, strapdown INS and the turntable can be seen in Figure 12. The specifications of the SGT-3 turntable are shown in Table 8. The IMU's three output axes (*x_b_*, *y_b_*, *z_b_*) paralleled to the turntable's inside frame, middle frame and outside frame respectively. At the start of each experiment, the three frames of the turntable initially turned to a static position for 20 seconds, *i.e.*, pitch angle 0°, roll angle 0°, yaw angle 180°, the start time of alignment *t*_0_ was chosen at the 10th second. Then all the three frames of the turntable started to sway with 5° in magnitude and 4 seconds in period. The total swaying time was 200 seconds. For each experiment, *t_k_*_1_ was set at the 100th second when the turntable enters the swaying mode. After finishing the swaying form, the turntable turned to the static mode again with the initial position for 20 seconds, *t_k_*_2_ was set at the 10th second in the static time, the pitch, roll and yaw angles of the turntable at this time were considered as references (the true values) to compare the results of the different alignment schemes. During the alignment process, a specific lever-arm between the strapdown INS and the turntable's frames should not be ignored. When the turntable was in swaying mode, the strapdown INS could accordingly experience a velocity component due to this lever-arm. And this velocity component can be regarded as the disturbing velocity in period form of 4 seconds too.

Fifty experiments were conducted. During the turntable experiments, the frequency of the disturbance could be regarded as 0.25 Hz (1/4 Hz), we set the IIR filter cutoff frequency *f_c_* as 0.2 Hz with order *N_IIR_* = 5, the transition-band of the FIR filter was [0.1 Hz, 0.2 Hz], stop-band attenuation *A_p_* = 40 *dB*, the order of FIR *N_FIR_* = 1418, *T_delay_* of the FIR was 7.09 seconds, and the parameters of the HMM-KF *Q_a_*, *R_a_*, *Q_g_*, *R_g_* were optimally set as follows:

$$\begin{array}{c} Q_{a}=\text{diag}{\left\{0.02*10^{-3}g,\;0.02*10^{-3}g\right\}}^{2},\;R_{a}=\text{diag}{\left\{100*10^{-4}g,\;100*10^{-4}g\right\}}^{2}\\ Q_{g}=\text{diag}{\left\{0.5*10^{-5}({}^{\circ}/h),\;0.5*10^{-5}({}^{\circ}/h)\right\}}^{2},\;R_{g}=\text{diag}{\left\{50*10^{-5}({}^{\circ}/h),\;50*10^{-5}({}^{\circ}/h)\right\}}^{2} \end{array}$$

**Figure 13.**
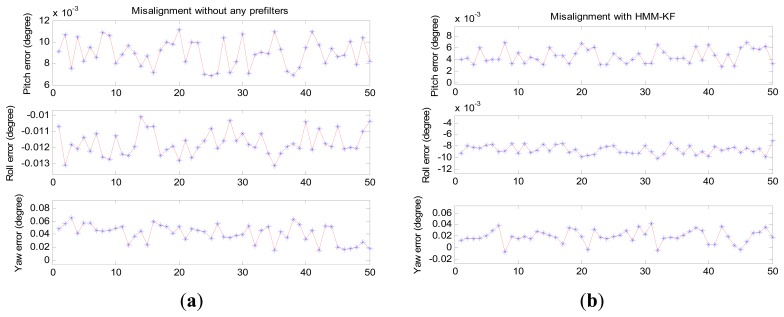

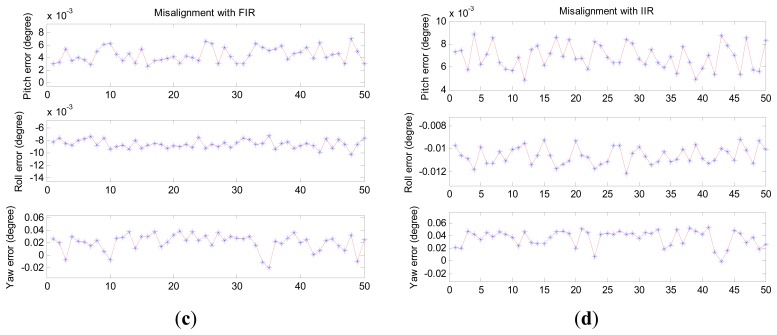
50 times turntable coarse alignment experiments results. (**a**) Misalignment without any prefilters; (**b**) Misalignment with HMM-KF; (**c**) Misalignment with FIR; (**d**) Misalignment with IIR (*N_IIR_* = 3).

Figure 13 provides the misalignment angles of pitch error *φ_e_*, roll error *φ_n_* and yaw error *φ_u_* of the 50 coarse alignment results using four different alignment schemes, *i.e.*, (a) alignment without any prefilters, only the inertial frame based alignment (IFBA) technique was adopted; (b) IFBA + HMM-KF aided alignment scheme; (c) IFBA + FIR aided alignment scheme; (d) IFBA + IIR aided alignment scheme. In accordance to Figure 13, Table 9 shows the mean and STD values of the misalignment angles using the four approaches. Figure 13 and Table 9 clearly indicate that all the four methods employing the IFBA technique can well accomplish the alignment process and get reasonable alignment results, this demonstrates the effectiveness of the IFBA for the in-motion or mooring alignment applications of strapdown INS. While the alignment methods with prefilters (HMM-KF, FIR and IIR filters) could more or less improve the alignment results than that without any prefilters. However, among the three frefilter aided alignment methodologies, the HMM-KF aided scheme can achieve the optimal alignment precisions, all the three misalignment angles are smaller than that of the IIR aided scheme, especially for the yaw misalignment angle *φ_u_*, precision increased by more than 0.01°. That is because different from the other two digital filter aided methods, the proposed HMM-KF could filter out the relatively high-frequency sensor noises and external disturbances both for the measurements of gyroscope and accelerometer.

Although the precision of the FIR aided method has almost the same level compared with that of the HMM-KF aided scheme. The computation time is obviously shorter for the HMM-KF aided scheme than that of the FIR aided method. In the 50 experiments with the alignment program written in C++ language, tested on an Intel Core 2 Due 1.94 GHz CPU, the average time for each FIR aided method is 4.03 seconds and 1.69 seconds for each HMM-KF aided method implementation.

As the experiments were conducted on the turntable, the external disturbances were constrained relatively in long periodical time (4 seconds), then the cutoff frequency *f_c_* was chosen smaller than 1/4 Hz, namely, 0.2 Hz in the experiments. However, as mentioned in the Introduction part, in practice, the frequencies of the external disturbances are mostly higher than 1/15 Hz during the mooring alignment. So accordingly, *f_c_* should be set smaller than 1/15 Hz, thus the orders and time delay of the digital filters will increase, and the corresponding results and the influence will be given and discussed in detail in the following mooring experiment section.

### 6.2. Mooring Experiment

**Figure 14.**
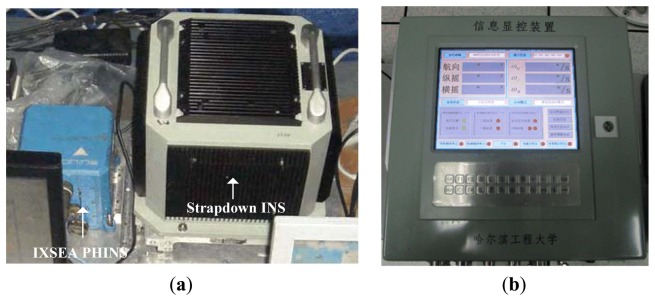
(**a**) Self-made strapdown INS, PHINS; (**b**) Information control panel.

The mooring experiment was conducted in the East Sea of China to validate the proposed alignment method. In this experiment, the ship was under mooring condition. A self-made IFOG based IMU was used for the experiment, the attitude reference was given by the PHINS from the company IXSEA, details of this system are described in [58]. The performance of the PHINS in GPS aided mode is as follows: both pitch and roll errors are less than 0.01°, heading error is less than 0.02°. The self-made IMU, PHINS and information control panel are shown in Figure 14. The fine alignment procedure was performed to estimate the misalignment angles by using a software package which is compiled by the Marine Navigation Research Institute of Harbin Engineering University. We carried out the alignment experiments three times. During the mooring experiments, the cutoff frequency *f_c_* was set as 0.03 Hz, the IIR order *N_IIR_* = 5, the FIR filter transition-band was [0.01 Hz, 0.05 Hz], stop-band attenuation *A_p_* = 40 *dB*, then the order of FIR *N_FIR_* = 3544, *T_delay_* of the FIR was 17.72 seconds, and accordingly the parameters of the HMM-KF *Q_a_*, *R_a_*, *Q_g_*, *R_g_* were optimal chosen as follows:

$$\begin{array}{c} Q_{a}=\text{diag}{\left\{0.003*10^{-3}g,\;0.003*10^{-3}g\right\}}^{2},\;R_{a}=\text{diag}{\left\{100*10^{-4}g,\;100*10^{-4}g\right\}}^{2}\\ Q_{g}=\text{diag}{\left\{2.5*10^{-5}({}^{\circ}/h),\;2.5*10^{-5}({}^{\circ}/h)\right\}}^{2},\;R_{g}=\text{diag}{\left\{50*10^{-5}({}^{\circ}/h),\;50*10^{-5}({}^{\circ}/h)\right\}}^{2} \end{array}$$

**Figure 15.**
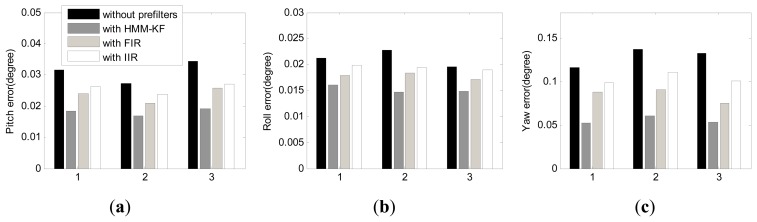
Comparison of the misalignment angles using the four alignment schemes. (**a**) Pitch error; (**b**) Roll error; (**c**) Yaw error.

**Figure 16.**
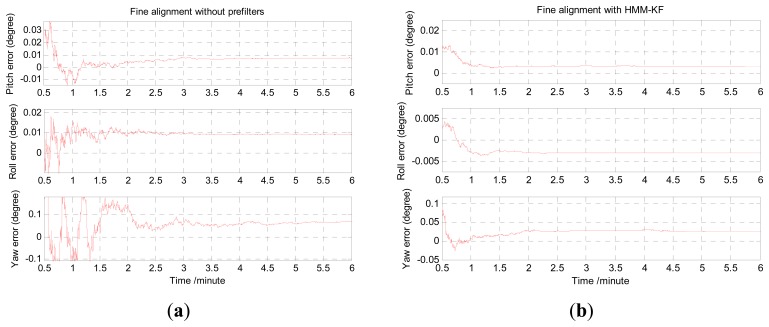

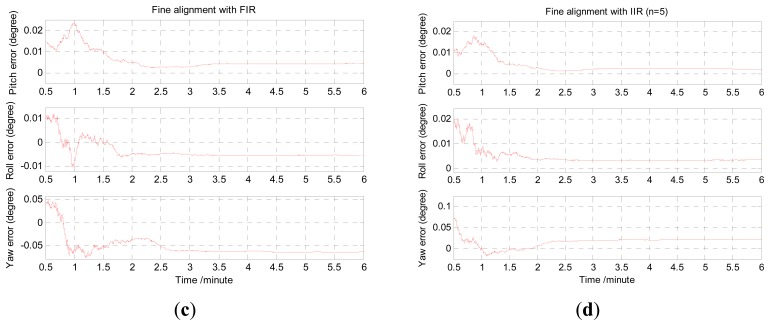
Estimation curves of the misalignment angles in fine alignment stage. (**a**) Fine alignment without any prefilters; (**b**) Fine alignment with HMM-KF; (**c**) Fine alignment with FIR; (**d**) Fine alignment with IIR (*N_IIR_* = 3).

The coarse and fine alignment time were set as 200 seconds and 6 minutes, respectively. The alignment results utilizing the above mentioned four schemes are depicted in histogram form in Figure 15. In accordance with Figure 15, the mean and STD values of the misalignment angles are shown in Table 10. It is clear from Figure 15 and Table 10 that the overall trends of the alignment results using the four schemes could also preferably validate the conclusions in Section 6.1. The misalignment angles of pitch errors and roll errors with HMM-KF are less than 0.02°, and yaw errors are less than 0.06°. Although the alignment results are less accurate than the results from the turntable experiments, while as the IMU was in mooing dynamic mode, these alignment results all the same primely fulfill the requirement and the accuracy of the ship's strapdown INS alignment under mooring condition. Moreover, compared with other three alignment schemes, the proposed HMM-KF aided scheme can not only help the Kalman filter in the fine alignment stage to converge faster, but also help to get more reliable misalignment angle estimates, especially for the yaw angle, that can be seen in Figure 16.

Figure 16(a–d) present the estimation curves of the misalignment angles in the fine alignment stage respectively utilizing the four different alignment schemes. For the convenience of clearly showing the convergence part of the estimation curves, the initial 30 seconds part was omitted. As depicted in Figure 16(a) and validated in ref. [11] that, under mooring condition, because the yaw error is in low observability, the estimation filters need relatively long time to converge to the true values when choosing the velocity errors as the only measurement vector to estimate the misalignment angles.

However, Figure 16(b) indicates that the HMM-KF aided scheme could shorten the estimation time to large extent, especially for the yaw error estimation. As can be seen in Figure 16(b) that the pitch and roll error estimations with HMM-KF are in about 1.5 minutes of convergence time instead of more than 3 minutes without any prefilters, and heading error convergence time with HMM-KF is about 2.5 minutes instead of more than 5 minutes without any prefilters. Figure 16(c) and (d) also show that FIR and IIR aided schemes could slightly shorten the estimation time to a certain extent. To our knowledge, for the self-alignment of strapdown INS under mooring condition, without using external information or equipments to augment the measurement vector of the system, such as GPS providing position information, the following three factors are able to improve the observability of the yaw misalignment angle using only the velocity errors as the measurement vector:

1. It has been well demonstrated that estimation process with large initial yaw (heading) error needs more time to converge than that with small initial yaw error [11,12,19,40]. That means that the precise coarse alignment result is an important factor which is to provide a reliable initial condition for the following fine alignment process. So the convergence speed of the yaw error estimate could decrease with a small initial coarse alignment result.
2. Also, through augmenting the signal to noise ratio (SNR) of gyroscope outputs could also shorten the yaw error estimation time because one reason why the yaw error estimate gets convergent slowly is that the earth rotational rate is too slow while the sensor noise is relatively two large under mooring dynamic conditions. Through utilizing some prefilter techniques (like the proposed HMM-KF) to preprocess the gyroscope outputs, the convergence speed and the accuracy of the yaw error estimate can also be improved.
3. In addition, the convergence speed of the yaw misalignment estimate could change by adjusting the estimated variance of the yaw azimuth. We have experientially and experimentally get the idea that in order to shorten the convergence time, the estimated variance of the yaw error should be initialized as a relatively large value.

### 6.3. Sea Experiment

**Figure 17.**
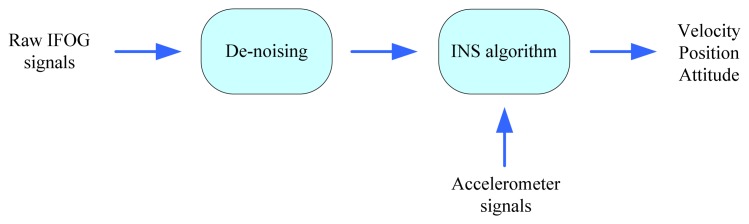
The de-noising of the IFOG signals before the navigation calculation stage.

A sea experiment was conducted to evaluate the online de-noising performance of the proposed HMM-KF. During the sea experiment, due to the dithering motion of ship engines, and commonly the inertial sensors could suffer from additional high-frequency random noises induced by external high dynamic motions, so the self-made medium-accuracy IMU (mainly for the self-made IFOG, marginally for the accelerometer) could experience not only the intrinsic sensor noises mentioned in Section 1, but also these additional random noises, thus bring some accumulative errors into the navigation solutions [37]. Some de-noising techniques have been used to filter out these noise components, such as Zu, *et al.* in [59,60] adopting the second generation wavelet transform method, and Li, *et al.* utilizing the different digital filters [61]. Here in this section, the proposed HMM-KF was adopted and tested for the on-line de-noising of the IFOG raw signals. The objective of the IFOG de-noising process is to improve the accuracy of the navigation solutions, *i.e.*, the velocity, position and attitude. Figure 17 illustrates the de-noising process.

As explained in Section 5 that the IIR digital filters are more reliable in real-time signal processing with relatively small time delay than that of the corresponding FIR filters. So in this experiment, the IIR filter was designed and utilized to compare the online de-noising performance with the proposed HMM-KF. At this time, the movements of the ship, whether caused by waves or the ship itself, are in relatively low frequencies. These movements are what we ask the IMU to measure, they are regarded as the useful signals for the navigation calculations. So the cutoff frequency *f_c_* to attenuate the noise components mentioned in last paragraph could be larger than those used in Section 6.1 and 6.2 for the alignment process. Here we set the IIR filter cutoff frequency *f_c_* as 5 Hz with the order *N_IIR_* = 3. Accordingly, the parameters *Q_a_*, *R_a_*, *Q_g_*, *R_g_* of the HMM-KF were optimally reset, and they are as follows:

$$Q_{g}=\text{diag}{\left\{2.5*10^{-5}({}^{\circ}/h),\;2.5*10^{-5}({}^{\circ}/h)\right\}}^{2},\;R_{g}=\text{diag}{\left\{50*10^{-5}({}^{\circ}/h),\;50*10^{-5}({}^{\circ}/h)\right\}}^{2}$$

**Figure 18.**
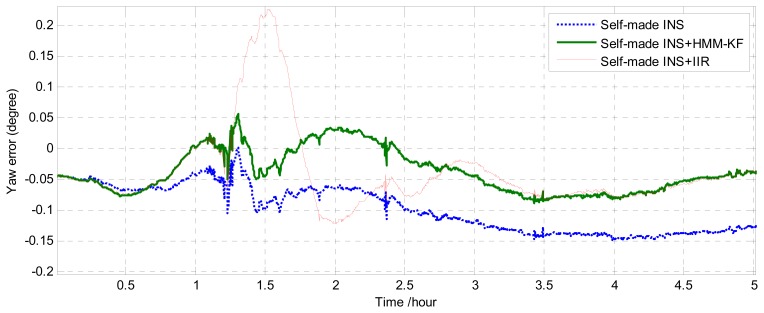
Comparison of the yaw (heading) error using the three schemes.

**Figure 19.**
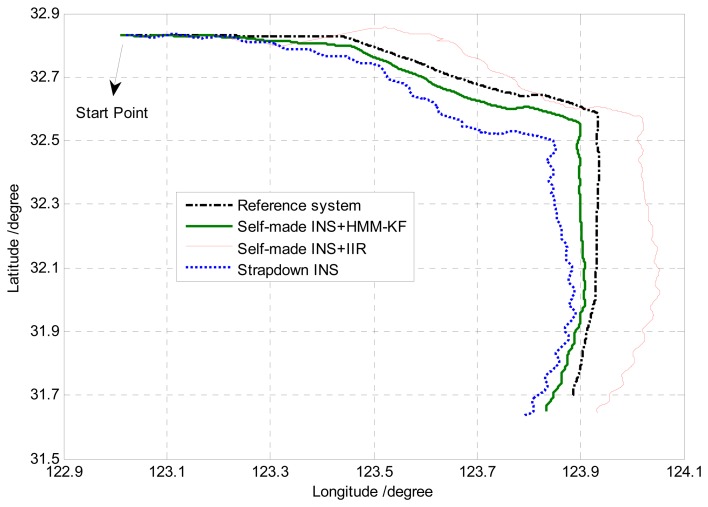
Trajectory comparison of the different navigation solutions.

After finishing the mooring alignment procedure of the strapdown INS in the dock, the ship sailed successively with speed change, heading change, manoeuvres, *etc*. The sea experiment lasted about 5 hours. The navigation data from the reference system (PHINS) and the self-made strapdown INS were collected, the navigation solutions (attitude, position) from PHINS were used as the reference solutions. The comparison of the yaw (heading) error utilizing the three different schemes (without any prefilters, with HMM-KF and with IIR filter) is shown in Figure 18. Trajectories derived from the three schemes and the reference system are illustrated in Figure 19.

Figures 18 and 19 clearly indicate that as compared with the scheme without any prefilters, the HMM-KF de-noising technique could obviously improve the accuracy of the heading and positioning solutions. In addition, as can be seen in Figure 18 that, because the IIR filter and the corresponding HMM-KF are designed to have the same de-noising performance, at most of the time in this experiment, the yaw errors of the two schemes are almost in the same level. However, it is also depicted in Figure 18 that there are noticeable vibrations for the three schemes between the time 1 *h* and 1.5 *h*, which were mainly caused by the ship's movements of rapid heading changes, manoeuvres, *et al.*, marginally caused by the limitations of the navigation algorithm itself. Besides, for the two schemes with HMM-KF and without any prefilters, both the yaw errors could quickly tend to be stable in the following time (1.5 *h*–).

As for the IIR de-noising scheme, these consecutive motions of heading changes and manoeuvres could cause severe time delay for the processed IFOG output signals, thus resulting in the maximum yaw error above 0.2° (at time 1.5 *h*), more than 2 hours' stabilizing time (1.5 *h*–3.5 *h*) and the divergence of the corresponding trajectory shown in Figure 19. These are all because that under high dynamic conditions, any small time delay between the original and filtered IFOG output signals will bring about some certain navigation errors for the strapdown INS, especially for those dynamic motions with frequencies close to the IIR filter's cutoff frequency *f_c_*, so the major drawback of using digital filters for this purpose is defining the proper filter cutoff frequency. On the contrary, the use of the proposed HMM-KF is much easier than the digital filters, as the former could merely cause time-delay issue once the optimal parameters of the specific HMM-KF are determined. So the results from the sea experiment indicate that the proposed HMM-KF could be used for the real-time de-noising in the navigation calculation process, and thus can improve the results of the navigation solutions.

Some remarks on the three experiments are made as follows:

1. Although the same IMU was used for the three experiments, the values of HMM-KF parameters *Q* and *R* are different. As we have not yet found a mathematical method to get the optimal HMM-KF parameters. So, to meet the requirements of the three different experiments, the choosing of *Q* and *R* are rather a matter of tuning based on the discussions and conclusions in Section 5. Each group of *Q* and *R* corresponds to a stable value of *K*_0_, and each *K*_0_ corresponds to a specific *f_c_*. Through experimentally adjusting the parameters *Q* and *R*, we could get the potential connections of the HMM-KF parameters (*Q* and *R*), *K*_0_, *f_c_* and the HMMKF time delay, such as the connections partially given in Tables 4, 5, 6 and 7. Based on these connections, we can feasibly get the parameters *Q* and *R* for each specific experiment.
2. It is worth mentioning that, although only one mooring experiment was conducted, all the results and conclusions are relevant to the one experiment, we still believe in the robustness of the proposed self-alignment method. As based on the researches about the topic “measurement of ship's instantaneous line motion under dynamic conditions” in [62–65], which were conducted by our group, that no matter under mooring or voyaging conditions, the external body dynamics of the ship are mainly caused by the sea waves. The relation between the testing platform properties, such as the size or mass of the testing ship, the water depth of the docks, *et al*. could merely cause the strong dynamics of the ship. However, this conclusion should and will be verified by the further experiments at different conditions.

## 7. Conclusions

When the ship is under mooring conditions, the IMU outputs from the ship-mounted strapdown INS not only contain the intrinsic sensor noise components but also experience the external random disturbances due to the movements of the ship, caused by the sea waves and wind waves. These mixed error components will result in both the inaccuracies of the alignment results and also the increasing of the alignment time.

This current work has presented a new robust scheme to solve the alignment problem of the ship's strapdown INS under mooring condition. The error components of accelerations and angular velocities will be effectively pre-filtered by using the proposed hidden Markov model based Kalman filter (HMM-KF). Different from the IIR and FIR digital filters aided techniques, as these methods will more or less cause the time-delay problems, the HMM-KF has good real-time ability at no cost of lowering the de-noising performance. After pre-processing the inertial sensors' outputs, we adopt the inertial frame based alignment (IFBA) method which can counteract and average the low-frequency periodical disturbed accelerations and angular velocities in our approach, in both the coarse and fine alignment procedures.

The turntable and mooring experiments results show that when the ship's strapdown INS is under mooring conditions, the proposed self-alignment scheme can make the initial attitude matrix of the strapdown INS built rapidly and accurately. Moreover, the sea experiment validate that the good de-noising performance of the proposed HMM-KF also can be applied in the navigation calculation process, making the navigation results calculated more accurately.

Although the effective de-noising results can be achieved by choosing appropriate initial values of HMM-KF parameters *Q_a_*, *R_a_*, *Q_g_*, *R_g_* However, different IMUs will have different statistical characteristics of the noise components. So the choices of the HMM-KF parameters are different from each IMUs. As there is no criterion to identify the optimal parameters of HMM-KF at the present time, only we can get the relatively optimal parameters is through using the experiment methods to realize.

Besides, some future works and improvements should be accomplished, they are as follows:

1. The Allan variance analysis will be used to exactly separate the random noise components and other error components, so we can effectively evaluate the de-nosing performances of the HMM-KF by choosing different filtering parameters.
2. Try to find an optimization method to adjusting the HMM-KF parameters. As we found that under high dynamic conditions, the de-noising results will more or less degenerate by using the parameters in static or in low dynamic conditions, so the adaptive adjusting and choosing of the HMM-KF parameters for the on-line de-noising applications under different conditions is also involved in our further studies.
3. Further works also include the expanding of other sensor suit to enhance the accuracy and rapidness of the mooring or in-motion alignment of ship's strapdown INS, such as GPS providing position information to enter the Kalman filter to bound the attitude errors in the alignment results during the fine alignment process.
4. Also, as we assume that the misalignment angles of coarse alignment results are in very small values, so during the fine alignment process, the state equation is regarded as the standard linear system. Future work should discuss the no-linear problem of the fine alignment process in case that the coarse alignment results are in large alignment angles. At that time, the utilizing of some complementary or no-linear filtering techniques is necessary.
5. Mooring alignment experiments should be conducted at different conditions and environments, and try to get some conclusions on the relation between the overall alignment performance and the above mentioned testing platform properties (*e.g*., the size or mass of the test ship, the water depth of the docks).

## Figures and Tables

**Table 1.** Specifications of the IFOG based IMU.

**Parameters**	**Gyroscope**	**Accelerometer**
Constant bias	0.01^o^/h	10^-4^g
Random noise	0.005^o^/h	$2\times 10^{-4}\text{g}/\sqrt{\text{Hz}}$
Dynamic range	±200^o^/s	±30g

**Table 2.** STD of accelerometer outputs with and without HMM-KF.

**Methods**	***f****_x_* [***m*/*s****^2^*]	***f****_y_* [***m*/*s****^2^*]	***f****_z_* [***m*/*s****^2^*]
STD without HMM-KF	0.0023	0.0019	0.0021
STD with HMM-KF	1.85e-4	6.93e-4	4.36e-4

**Table 3.** STD of gyroscope outputs with and without HMM-KF.

**Methods**	***ω****_x_* [**^o^/*h***]	***ω****_y_* [**^o^/*h***]	***ω****_z_* [**^o^/*h***]
STD without HMM-KF	3.2836	7.5493	6.0536
STD with HMM-KF	0.2823	0.7261	0.6597

**Table 4.** Values of *K*_0_, *f_c_* and *N_FIR_* with different *q* and *r*.

**Parameters**	***K*_0_**	***f****_c_* [**Hz**]	***N****_FIR_* [**Minimum order**]	

			***A****_s_***= 40*dB***	***A****_s_***= 60*dB***
*r*_1_ = 500, *q* = 0.5	0.0107	0.9836	1678	2255
*r*_2_ = 1,000, *q* = 0.5	0.0055	0.4914	1712	2319
*r*_3_ = 1,500, *q* = 0.5	0.0028	0.2139	1726	2420

*q*_1_ = 0.1, *r* = 100	0.0102	0.9831	1681	2257
*q*_2_ = 0.05, *r* = 100	0.0052	0.4912	1712	2319
*q*_3_ = 0.02, *r* = 100	0.0020	0.1964	1731	2454

**Table 5.** Values of *K*_0_, *f_c_* and *N_FIR_* with different *m* and *n*.

**Parameters**	***K*_0_**	***f****_c_* [**Hz**]	***N****_FIR_* [**Minimum order**]	

			***A****_s_***= 40*dB***	***A****_s_***= 60*dB***
*n*_1_ = 200, *m* = 2	0.0103	0.9831	1681	2257
*n* = 500, *m* = 2	0.0043	0.3927	1721	2335
*n*_3_ = 1,000, *m* = 2	0.0021	0.1965	1732	2454

*m*_1_ = 0.5, *n* = 50	0.0102	0.9831	1681	2257
*m*_2_ = 0.25, *n* = 50	0.0054	0.4914	1713	2319
*m*_3_ = 0.125, *n* = 50	0.0025	0.2138	1726	2420

**Table 6.** Comparison of the time delay (in samples) of the z axis acceleration using the three filters.

**Parameters**	**HMM-KF** [**samples**]	**5 order IIR** [**Max. samples**]	**10 order IIR** [**Max. samples**]	**FIR*A****_s_*= **40*dB*** [**samples**]
*r*_1_ = 500, *q* = 0.5	5	84	199	839
*r*_2_ = 1,000, *q* = 0.5	18	167	398	856
*r*_3_ = 1,500, *q* = 0.5	41	384	913	863

*q*_1_ = 0.1, *r* = 100	1	84	197	838
*q*_2_ = 0.05, *r* = 100	4	164	398	855
*q*_3_ = 0.02, *r* = 100	6	428	1016	867

**Table 7.** Comparison of the time delay (in samples) of the z axis angular rate using the three filters.

**Parameters**	**HMM-KF** [**samples**]	**5 order IIR** [**Max. samples**]	**10 order IIR** [**Max. samples**]	**FIR * A****_s_* **= 40*dB*** [**samples**]
*n*_1_=200, *m* = 2	10	84	199	841
*n*_2_ = 500, *m* = 2	42	208	486	862
*n*_3_ = 1,000, *m* = 2	70	411	991	866

*m*_1_ = 0.5, *n* = 50	1	84	199	841
*m*_2_ = 0.25, *n* = 50	3	165	391	857
*m*_3_ = 0.125, *n* = 50	4	384	914	863

**Table 8.** Specifications of the SGT-3 turntable.

**Parameters**	**Precision**
Position resolution	±3” (1° = 60′=3600”)
Position range	±360°

Angular rate resolution	0.001°/h
Angular rate range	± 150°/s

**Table 9.** Mean and STD of the misalignment angles using the four alignment schemes.

**Alignment Schemes**	***φ****_e_* [**degree**]	***φ****_n_* [**degree**]	***φ****_u_* [**degree**]
Mean without Prefilters	0.0095	-0.0113	0.0418
Mean with HMM-KF	0.0049	-0.0085	0.0197
Mean with FIR	0.0053	-0.0087	0.0204
Mean with IIR (*N_IIR_* = 3)	0.0073	-0.0106	0.0317

STD without Prefilters	0.0027	9.166e-4	0.0158
STD with HMM-KF	0.0012	6.549e-4	0.0099
STD with FIR	0.0013	6.758e-4	0.0120
STD with IIR (*N_IIR_* = 3)	0.0025	8.692e-4	0.0086

**Table 10.** Mean and STD of the misalignment angles using the four alignment schemes.

**Alignment Schemes**	***φ****_e_* [**degree**]	***φ****_n_* [**degree**]	***φ****_u_* [**degree**]
Mean without Prefilters	0.0311	0.0212	0.1287
Mean with HMM-KF	0.0181	0.0152	0.0554
Mean with FIR	0.0236	0.0178	0.0871
Mean with IIR (*N_IIR_* = 3)	0.0257	0.0194	0.1037

STD without Prefilters	0.0036	0.0016	0.0111
STD with HMM-KF	0.0012	0.0014	0.0047
STD with FIR	0.0023	0.0019	0.0033
STD with IIR (*N_IIR_* = 3)	0.0017	0.0005	0.0064

## Appendix

### A. The derivation of $C_{i_{b0}}^{i}$

After the alignment process gets started, the projection of the pure gravity vector in the *i* frame can be expressed as follows:

(A1) $$g^{i}=\left[\begin{array}{c} -g\cos L\cos[\lambda +\omega_{ie}(t-t_{0})]\\ -g\cos L\sin[\lambda +\omega_{ie}(t-t_{0})]\\ -g\sin L \end{array}\right]$$

Then the velocity of strapdown INS in the *i* frame can be calculated by the integration of Equation (A1) from time *t*_0_ to *t_k_*:

(A2) $$V^{i}(t_{k})=\left[\begin{array}{c} g\cos L\{\sin[\lambda +\omega_{ie}(t_{k}-t_{0})]-\sin\lambda \}/\omega_{ie}\\ g\cos L\{\cos\lambda -\cos[\lambda +\omega_{ie}(t_{k}-t_{0})]\}/\omega_{ie}\\ g\sin L(t_{k}-t_{0}) \end{array}\right]$$

Under mooring condition, the measurements of specific force provided by the accelerometers in the *b* frame can be expressed by:

(A3) $$f^{b}=-g^{b}+\delta a^{b}+a_{\text{noise}}^{b}$$

where *g^b^* is the local gravity expressed in the *b* frame, *δa^b^* is the external disturbed components, $a_{\text{noise}}^{b}$ is the random sensor noise. Ignoring the sensor noise $a_{\text{noise}}^{b}$ and transforming *f^b^* from the *b* frame into the *i*_*b*0_ frame yields:

(A4) $$f^{i_{b0}}=C_{b}^{i_{b0}}f^{b}=-C_{b}^{i_{b0}}g^{b}+C_{b}^{i_{b0}}\delta a^{b}$$

The integration of Equation (A4) from time *t*_0_ to *t_k_* can be described as follows:

(A5) $$\begin{array}{ll} V^{i_{b0}} & =\int_{t_{0}}^{t_{k}}-C_{b}^{i_{b0}}g^{b}dt+\int_{t_{0}}^{t_{k}}C_{b}^{i_{b0}}\delta a^{b}dt\\ & =-C_{i}^{i_{b0}}\int_{t_{0}}^{t_{k}}g^{i}dt+\int_{t_{0}}^{t_{k}}C_{b}^{i_{b0}}\delta a^{b}dt\\ & =C_{i}^{i_{b0}}V^{i}+\Delta V^{i_{b0}} \end{array}$$

where $V^{i}=-\int_{t_{0}}^{t_{k}}g^{i}dt$, $\Delta V^{i_{b0}}=\int_{t_{0}}^{t_{k}}C_{b}^{i_{b0}}\delta a^{b}dt$, as the external disturbance *δa^b^* is mainly caused by the sea waves which could be decomposed into many small components in periodic form, that is to say most of the disturbance may change periodically. Then after the integration of *ba^b^*, Δ*V*^*ib*0^ could be expressed approximately as zero, namely $\Delta V^{i_{bo}}=\int_{t_{0}}^{t_{k}}C_{b}^{i_{b0}}\delta a^{b}dt\approx 0$. Substituting Δ*V*^*i*^*b*0^^ in Equation (A5) gives:

(A6) $$V^{i_{b0}}=C_{i}^{i_{b0}}V^{i}$$

From Equation (A6) we can get the different values of *V^i^* at different time, so at time *t_k_*_1_ and *t_k_*_2_ (*t*_0 <_ *t_k_*_1 <_ *t_k_*_2_) we have:

(A7) $$\{\begin{array}{c} V^{i_{b0}}(t_{k1})=C_{i}^{i_{b0}}V^{i}(t_{k1})\\ V^{i_{b0}}(t_{k2})=C_{i}^{i_{b0}}V^{i}(t_{k2}) \end{array}$$

Combining Equations (A6) and (A7) and rearranging yields:

(A8) $$C_{i_{b0}}^{i}={\left[\begin{array}{c} {\left[V^{i}(t_{k1})\right]}^{T}\\ {\left[V^{i}(t_{k2})\right]}^{T}\\ {\left[V^{i}(t_{k1})\times V^{i}(T_{k2})\right]}^{T} \end{array}\right]}^{-1}\left[\begin{array}{c} {\left[V^{i_{b0}}(t_{k1})\right]}^{T}\\ {\left[V^{i_{b0}}(t_{k2})\right]}^{T}\\ {\left[V^{i_{b0}}(t_{k1})\times V^{i_{b0}}(t_{k2})\right]}^{T} \end{array}\right]$$

### B. Connection between K_0_ and f_c_

Performing Z-transform on Equation (27) results in the system's transfer function:

(B1) $$H(Z)=\frac{Y(Z)}{X(Z)}=\frac{K_{0}\cdot Z}{1-(1-K_{0})}$$

For the convenience of system frequency responses analysis, we transform Equation (B1) into S-domain:

(B2) $$H(e^{i\omega T})=\frac{K_{0}}{a-ib}=\frac{K_{0}}{a^{2}+b^{2}}(a+bi)$$

where *a* = 1−(1−K_0_) cos (*ωT*), *b* = (1−*K_0_*) sin (*ωT*). From Equation (B2) we can get the amplitude-frequency characteristic function |*H* (*e^iωT^*) of the proposed HMM-KF:

(B3) $$\left|H(e^{i\omega T})\right|=\frac{K_{0}}{\sqrt{a^{2}+b^{2}}}=\frac{K_{0}}{\sqrt{{1-2(1-K_{0})\cos(\omega T)+(1-K_{0})}^{2}}}$$

When *ω*=0, |*H* (*e^iωjT^*)| can get the maximum value, namely 1. As the cutoff frequency is usually d efined to be where the amplitude is reduced to $1/\sqrt{2}=0.707$ (*i.e.*, −3 dB). Suppose *ω_f_* is the frequency when the value of |*H ( e^iωT^*)| decline to $\frac{1}{\sqrt{2}}$, which satisfies:

(B4) $$\frac{K_{0}}{\sqrt{1-2(1-K_{0})\cos(\omega_{f}T_{s})+{\left(1+K_{0}\right)}^{2}}}=\frac{1}{\sqrt{2}}.1$$

Expanding Equation (B4), we have the circle frequency *ω_f_*[57]:

(B5) $$\omega_{f}=\frac{1}{T_{s}}\arccos\frac{2-2K_{0}-{\left(K_{0}\right)}^{2}}{2(1-K_{0})}$$

Then the system's real cutoff frequency *f_c_* is:

(B6) $$f_{c}=\frac{\omega_{f}}{2\pi }\cdot f_{s}=\frac{1}{2\pi {\left(T_{s}\right)}^{2}}\arccos\frac{2-2K_{0}-{\left(K_{0}\right)}^{2}}{2(1-K_{0})}$$

where *f_s_* and *T_s_* are sampling frequency and sampling period respectively.
